# Do Transformers and CNNs Learn Different Concepts of Brain Age?

**DOI:** 10.1002/hbm.70243

**Published:** 2025-06-09

**Authors:** Nys Tjade Siegel, Dagmar Kainmueller, Fatma Deniz, Kerstin Ritter, Marc‐Andre Schulz

**Affiliations:** ^1^ Department of Psychiatry and Neurosciences Charité – Universitätsmedizin Berlin (Corporate Member of Freie Universität Berlin, Humboldt‐Universität zu Berlin, and Berlin Institute of Health) Berlin Germany; ^2^ Max‐Delbrueck‐Center for Molecular Medicine in the Helmholtz Association Berlin Germany; ^3^ Helmholtz Imaging Berlin Germany; ^4^ Digital Engineering Faculty of the University of Potsdam Potsdam Germany; ^5^ Bernstein Center for Computational Neuroscience Berlin Germany; ^6^ Faculty of Electrical Engineering and Computer Science Technische Universität Berlin Berlin Germany; ^7^ Hertie Institute for AI in Brain Health University of Tübingen Tübingen Germany

## Abstract

“Predicted brain age” refers to a biomarker of structural brain health derived from machine learning analysis of T1‐weighted brain magnetic resonance (MR) images. A range of machine learning methods have been used to predict brain age, with convolutional neural networks (CNNs) currently yielding state‐of‐the‐art accuracies. Recent advances in deep learning have introduced transformers, which are conceptually distinct from CNNs, and appear to set new benchmarks in various domains of computer vision. Given that transformers are not yet established in brain age prediction, we present three key contributions to this field: First, we examine whether transformers outperform CNNs in predicting brain age. Second, we identify that different deep learning model architectures potentially capture different (sub‐)sets of brain aging effects, reflecting divergent “concepts of brain age”. Third, we analyze whether such differences manifest in practice. To investigate these questions, we adapted a Simple Vision Transformer (sViT) and a shifted window transformer (SwinT) to predict brain age, and compared both models with a ResNet50 on 46,381 T1‐weighted structural MR images from the UK Biobank. We found that SwinT and ResNet performed on par, though SwinT is likely to surpass ResNet in prediction accuracy with additional training data. Furthermore, to assess whether sViT, SwinT, and ResNet capture different concepts of brain age, we systematically analyzed variations in their predictions and clinical utility for indicating deviations in neurological and psychiatric disorders. Reassuringly, we observed no substantial differences in the structure of brain age predictions across the model architectures. Our findings suggest that the choice of deep learning model architecture does not appear to have a confounding effect on brain age studies.

## Graphical abstract

(a) Modern shifted window transformer and well‐established ResNet50 CNN predicted brain age with on‐par accuracy. (b) Effect sizes between prediction errors (brain age gaps, BAGs) of patients and matched controls were similar for CNN and transformers across neurological and psychiatric diseases, yielding no indication that different model architectures base their predictions on different brain aging effects.

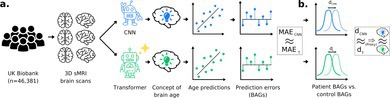

## 1 Introduction

The brain undergoes structural changes while aging (MacDonald and Pike 2021), leading to reduced cognitive function and increased risk of neurodegenerative disorders (Peters 2006; Farooqui and Farooqui 2009). The rate of these age‐related changes appears to be influenced by the presence of disease (Anderton 1997), lifestyle (Peters 2006), and environmental factors (Esiri 2007).

Brain age prediction estimates biological age using machine learning (ML) techniques applied to neuroimaging data. These prediction models are typically trained on healthy cohorts, ensuring that the model learns the amount of aging considered normal for healthy subjects (Feng et al. 2020; Dinsdale et al. 2021; Kolbeinsson et al. 2020). The difference between brain‐predicted age and chronological age (brain age gap, BAG) (Ballester et al. 2023; Chen et al. 2022; Man et al. 2021) has emerged as a valuable biomarker. Studies have shown elevated BAGs in patients with various psychiatric and neurological disorders, including Alzheimer's disease (AD), Parkinson's disease (PD), multiple sclerosis (MS), mild cognitive impairment (MCI), major depression (MD), schizophrenia, and bipolar disorder (BD) (Beheshti et al. 2020; Cole et al. 2020; Eickhoff et al. 2021; Bashyam et al. 2020; Nenadić et al. 2017; Kaufmann et al. 2019). Additionally, elevated BAGs have been linked to markers of poor health such as obesity, high blood pressure, and diabetes (Wrigglesworth et al. 2021). This elevation in BAGs is thought to arise from an overlap between the effects of aging, the secondary neurobiological effects of diseases, and poor general health (Cole and Franke 2017). The accumulating evidence linking BAGs with various health‐related factors and neurological and mental diseases has established BAGs as promising individualized biomarkers of structural brain health (Cole and Franke 2017).

Research suggests that accurate brain age models are essential to provide useful biomarkers (Hahn et al. 2021; Peng et al. 2021; Cole 2020; Tanveer et al. 2023; Niu et al. 2020). In this context, deep learning with convolutional neural networks (CNNs) has yielded the most accurate age predictions to date (Peng et al. 2021; Gong et al. 2021; Leonardsen et al. 2022). These deep learning model architectures can operate on minimally processed neuroimaging data, primarily voxel‐wise structural magnetic resonance imaging (sMRI) brain images (Feng et al. 2020; Dinsdale et al. 2021; Lee et al. 2022; Peng et al. 2021; Leonardsen et al. 2022). By using voxel‐wise input images, CNNs can learn to model complex visual features of brain aging from the ground up.

A recent innovation in deep learning architectures has been the development of transformer model architectures Vaswani et al. (2017), such as vision transformers (Dosovitskiy et al. 2021). While CNNs are built on specific assumptions about input images, such as spatial proximity of relevant information (LeCun et al. 2010), vision transformers have minimal vision specific inductive biases. They can integrate information from distant regions of the input, enabling the creation of visual features not constrained by spatial locality. Despite vision transformers' greater flexibility in forming spatially independent features requiring substantially larger amounts of training samples (Dosovitskiy et al. 2021), these model architectures seem to surpass CNNs' benchmarks in various domains of computer vision, including image classification (Dosovitskiy et al. 2021), semantic segmentation (Xie et al. 2021), and object detection (Liu et al. 2022). Given the success of vision transformers, two key questions emerge: Can transformers be utilized to make brain age predictions more accurate? And—since it is conceivable that characterizing only a small subset of aging effects in the brain is sufficient for accurately predicting age—do conceptually distinct deep learning model architectures learn different “concepts of brain age” (see Section 3.1)? As the mechanism by which CNNs and transformers learn visual features fundamentally differs, CNNs for brain age predictions could learn to characterize one subset of brain aging effects, while transformers could learn to characterize another.

If different deep learning model architectures attend to different concepts of brain age, this would pose multiple concerns for the brain age research paradigm: First, different model architectures could confound the results of prior studies, as different concepts of brain age could identify different disease‐related patterns. Comparing how informative BAGs are to diseases and health‐related factors would become challenging if different model architectures are employed, even in similar cohorts. Second, selecting a model architecture for brain age prediction would become increasingly complicated. For instance, one brain age concept could encompass a broad range of disease patterns, while others entail only a few. Hence, the selection of a model architecture would require measures of clinical utility rather than solely relying on model accuracy, despite the latter being the current common practice (Han et al. 2022; Baecker et al. 2021; Niu et al. 2020; Kuo et al. 2021; Amoroso et al. 2019). Third, if different brain age concepts inform on specific diseases, the role of BAGs as general brain health biomarkers, previously highlighted by Cole and Franke (2017), would require reevaluation. From a practical perspective, identifying which model architecture corresponds to which brain age concept would be essential, as BAGs might indicate specific diseases rather than general brain health.

To investigate whether different deep learning model architectures learn different concepts of brain age or achieve different levels of prediction accuracy, we adapted two popular transformer architectures: the simple vision transformer (sViT) (Beyer et al. 2022) and the shifted window transformer (SwinT) (Liu et al. 2021) for age prediction from 3D T1‐weighted sMRI brain scans. For comparison, we trained a ResNet He et al. (2016), a CNN architecture widely used in brain age prediction (Fisch et al. 2021; Jónsson et al. 2019; Kolbeinsson et al. 2020; Ballester et al. 2021; Shah et al. 2022; Hu et al. 2023). The selected model architectures span from low vision‐specific inductive bias and high flexibility in learning spatially independent features (sViT), to intermediate bias and flexibility (SwinT), to high bias and low flexibility (ResNet) (see Section 4.4).

**FIGURE 1:**
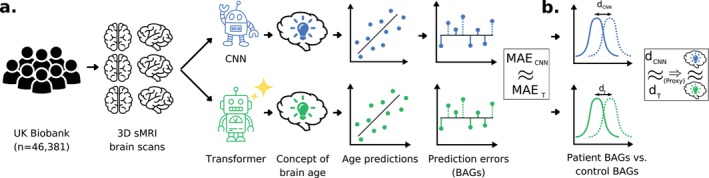
Overview of workflow and results: (a) We used 46.381 structural magnetic resonance imaging (sMRI) brain scans from the UK Biobank (UKBB) to train and evaluate a convolutional neural network (CNN; 3D ResNet50) and two transformers (3D simple vision transformer; sViT; 3D shifted window transformer; SwinT) for brain age prediction. Mean absolute errors (MAEs) for held‐out healthy subjects were nearly identical for ResNet (2.66 years) and SwinT (2.67 years). We define the term “concept of brain age” as the distinct brain aging effects identified by a brain age model and the way these aging effects are synthesized into scalar predictions. (b) Effect sizes between prediction errors (brain age gaps; BAGs) of patients and matched controls were similar for CNN and transformers across neurological‐ and psychiatric diseases, yielding no indication that different model architectures rely on meaningfully different concepts of brain age for their predictions.

We systematically investigate whether ResNet, sViT, and SwinT attend to meaningfully different concepts of brain age by examining two key aspects (see Section 3.3): differences in their predictions and clinical utility (ability to inform on neurological and psychiatric diseases, health‐related factors). These aspects serve as proxies for differences in how “brain age” is characterized by either model architecture. Divergent predictions and clinical utility across model architectures would suggest variations in the model architectures' concepts of brain age. To measure clinical utility we concentrate on diseases commonly examined in brain age studies, namely PD (Eickhoff et al. 2021), MS (Cole et al. 2020), epilepsy (Sone et al. 2021), alcohol use disorder (AUD) (Bøstrand et al. 2022), BD (Hajek et al. 2019), and psychotic disorders (Ballester et al. 2022). Additionally, we examine factors associated with brain health, specifically fluid intelligence, reaction time, trailmaking interval (Smith et al. 2019), tobacco consumption (Franke et al. 2013), mobile phone usage (Thomée 2018), TV consumption (Dougherty et al. 2022), systolic blood pressure (Smith et al. 2019), grip strength (Carson 2018), and body mass index (BMI) (Ward et al. 2005). An overview of our workflow and results is displayed in Figure 1.

## 2 Related Work

Previous works have been concerned with technical aspects of brain age prediction, such as bias correction (Beheshti et al. 2019; de Lange and Cole 2020; Zhang et al. 2023; Liang et al. 2019), performance metrics (de Lange et al. 2022), and prediction accuracy of different ML models (Valizadeh et al. 2017; Baecker et al. 2021; Lam et al. 2020). Some model comparisons have extended beyond these aspects to examine reliability measures (Bacas et al. 2023; Dörfel et al. 2023), aggregate measures of clinical utility (Lee et al. 2021; More et al. 2023; Xiong et al. 2023; Lee 2023; Beheshti et al. 2021), and general feature importance (Ball et al. 2021; Han et al. 2022). Specifically, for CNNs, several studies have investigated general feature importance (Lee et al. 2022; Hepp et al. 2021; Levakov et al. 2020; Hofmann et al. 2022).

However, to the best of our knowledge, no previous work has explicitly considered that the fundamental “concept of brain age” could differ between ML models, nor have any studies included conceptually different deep learning model architectures.

Furthermore, we believe this work represents the first application of cutting‐edge transformers for brain age prediction using minimally processed 3D sMRI data. Previous transformer applications in this domain have been limited to: 2D image slices (Zhang and Jiang 2024; Qodrati et al. 2023), CNN‐based feature encoder combinations for multi‐scale (He, Grant, et al. 2021) and multi‐modal fusion (Zhao et al. 2024; Cai et al. 2022; He, Pereira, et al. 2021), global information pathway augmentation (Hu et al. 2022), or to refine CNN‐extracted features from 2D image slices (Jun et al. 2021).

## 3 Theory

To frame our study, we introduce three key constructs. First, we define the “concept of brain age,”^1^ acknowledging the potential existence of multiple brain ages and the need for more precise terminology. Second, we discuss the “clinical utility” of these concepts, aiming to quantify their usefulness in clinical settings. Third, we explore internal representations in brain age models that encode these varied concepts and discuss methods to probe these representations.

### 3.1 Different Concepts of Brain Age

In the past, brain age has generally been regarded as a uniform concept, yet different models may accurately predict age while relying on different brain aging effects. Such aging effects may include enlargement of ventricles, cortical atrophy (especially in frontal areas) and, atrophy of subcortical gray matter structures, with regions like the hippocampus showing disproportionate changes (MacDonald and Pike 2021). To distinguish different combinations of brain aging effects, we introduce the term “concept of brain age”, referring to the aging effects characterized for age prediction (e.g., ventricle enlargement and frontal lobe atrophy), and how these aging effects are combined into a scalar prediction (e.g., ventricle enlargement weights stronger than frontal lobe atrophy). A concept of brain age differs from the features learned by a brain age model, since different features can be learned to represent the same aging effect (e.g., hippocampus intensity or volume could reflect hippocampal atrophy), while a brain age concept captures how different aging effects are combined.

Brain age concepts may vary in both scope and specificity, reflecting uncertainty about whether accurate predictions require broad or narrow ranges of aging indicators. This uncertainty arises from the likely redundancy in age‐related information carried by different brain aging effects. Bethlehem et al. (2022) demonstrated this by computing detailed normative trajectories for various regional brain structure volumes across the lifespan. In principle, (non‐linear) models could learn to predict age from any of these trajectories or their combinations. The specific aging effects captured may depend on model architecture, initialization, training data volume, and model capacity.

Rather than merely representing different measurement approaches, distinct brain age concepts fundamentally define brain age's nature. This becomes evident when considering that diseases and health‐related factors show regional preferences (Geng et al. 2006; Raz and Rodrigue 2006; Dekker et al. 2021; Gómez‐Apo et al. 2021; Gallinat et al. 2006). For example, hypertension appears to accelerate hippocampal shrinkage (Raz et al. 2005), suggesting models based on hippocampal atrophy may show elevated brain age in hypertension, while models focused on unrelated aging effects may not. Though ideally brain age concepts would encompass holistic aging effects, the redundancy in age‐related information makes it uncertain whether current models achieve such comprehensiveness.

### 3.2 Clinical Utility

To evaluate brain age concepts' practical value, we define “clinical utility” as a model's ability to inform on various diseases and health‐related phenotypes. We assess this through two approaches: examining BAG sensitivity to differences between healthy individuals and those with neurological and mental disorders (Cole et al. 2020; Bashyam et al. 2020; Kaufmann et al. 2019), and evaluating BAGs' predictive power for health‐related phenotypes (Cole 2020; Steffener et al. 2016; Lee 2023).

### 3.3 Probing Differences in Model Architectures' Concepts of Brain Age

Our goal is to determine whether different deep learning architectures develop distinct brain age concepts. While directly examining prediction‐relevant features might seem ideal, current methods for analyzing complex, non‐linear deep learning architectures face critical, unresolved reliability challenges (Kindermans et al. 2019; Adebayo et al. 2018; Sundararajan et al. 2017; Hooker et al. 2019; Dombrowski et al. 2019). Instead, we use “clinical utility” as a proxy to meaningful differences in concepts of brain age, examining how models' BAGs respond to diseases and health‐related phenotypes.

This approach provides insights into brain age concept differences because these conditions interact specifically with regional aging effects. For example, hypertension has been linked to accelerated hippocampal shrinkage (Raz et al. 2005); PD patients have shown significant atrophy in the pallidum Geng et al. (2006); tobacco use has appeared to reduce gray matter volume and density in the frontal, occipital, and temporal lobes Gallinat et al. (2006); MS has been associated with cerebellar and thalamic atrophy alongside white matter lesions (Dekker et al. 2021); and obesity has been related to gray matter loss in the frontal and temporal regions, basal nuclei, and cerebellum (Gómez‐Apo et al. 2021). Thus, brain age concepts may differ in their interaction with disease‐ or behavior‐related alterations based on their underlying aging effects.

## 4 Material and Methods

### 4.1 Participants

Our study is based on the UKBB, an ongoing prospective biomedical data collection initiative (Sudlow et al. 2015). Specifically, we used data from 46,381 individuals (53% female, age range 44‐83, age mean 64.26, age standard deviation 7.75), for whom T1‐weighted sMRI brain scans were available at the time of writing. We divided subjects into a normative cohort with no diagnoses in ICD‐10 category F (mental and behavioral disorders) and G (diseases of the nervous system), and a patient cohort including all diagnosis in category F and G. To determine how sensitive BAGs are to neurological and psychiatric disorders, we focus on disorders that are frequently studied in the context of brain age research: patients with PD (Eickhoff et al. 2021), MS (Cole et al. 2020), epilepsy (Sone et al. 2021), AUD (Bøstrand et al. 2022), BD (Hajek et al. 2019) and psychotic disorders^2^ (Ballester et al. 2022) in conjunction with controls from the normative cohort. We selected controls by matching normative subjects to the disease cohorts for each diagnosis using propensity score matching, while controlling for sex, age, education level, household income, the Townsend deprivation index, and genetic principal components, as described in (Schulz, Siegel, et al. 2024). The remainder of the normative cohort was used for model training. Patients who were not used to measure BAGs' sensitivity to diseases (patients were also not used for model training) were used to validate the hyperparameters of the model architectures, which led to the following set sizes: $n_{\text{train}}=27,538$, $n_{\mathrm{val}}=16,499$, $n_{\text{control}/\text{test}}=1,172$.

### 4.2 sMRI Data

We used minimally preprocessed 1 mm T1‐weighted sMRI brain scans provided by the UKBB. The images were skull‐stripped with the UKBB‐provided brain mask, linearly registered on MNI152 with the UKBB‐provided transformation matrices, and center‐cropped, resulting in a final resolution of 160 × 192 × 160, following standard preprocessing approaches (Peng et al. 2021; Leonardsen et al. 2022; Fisch et al. 2021; Kolbeinsson et al. 2020).

### 4.3 Target Phenotypes

In addition to the sMRI data, we used phenotypic data from the UKBB. Specifically, the UKBB provides information on ICD‐10 diagnosis in terms of first occurrence dates, and we assigned disease labels if the first occurrence date was before the date on which the sMRI data were collected. The mappings from diseases to UKBB fields are shown in the Table S1. To analyze BAG informativeness for brain health factors, we examined UKBB variables across three domains: cognitive performance (fluid intelligence, reaction time, trailmaking interval; Smith et al. 2019), lifestyle choices (tobacco consumption; Franke et al. 2013, mobile phone usage; Thomée 2018, TV consumption; Dougherty et al. 2022) and biomedical condition (systolic blood pressure; Smith et al. 2019, grip strength; Carson 2018, BMI; Ward et al. 2005). The mapping of each variable to the UKBB field number is provided in the Table S2.

### 4.4 Deep Learning Model Architectures

#### 4.4.1 3D ResNet50

As CNN architecture, we used a ResNet50 (He et al. 2016), adapted to 3D input (Hara et al. 2018). ResNet is a well‐known standard architecture in computer vision and is widely used in brain age prediction (Fisch et al. 2021; Jónsson et al. 2019; Kolbeinsson et al. 2020; Ballester et al. 2021; Shah et al. 2022; Hu et al. 2023). Conceptually, a simpler form of the ResNet is the VGG (Simonyan and Zisserman 2024) (or in its shallow form, the SFCN; Peng et al. 2021), which some brain age studies employ, too (Tanveer et al. 2023). In brief, the main component of ResNet (and VGG) is the convolutional layer, which incorporates convolutional filters that slide across the input image and combine local image information to create visual features such as edges or shapes. In our experiments, we used a conventional PyTorch implementation^3^ of the 3D ResNet50 (Hara et al. 2018), with a total number of 46.2 million trainable parameters.

#### 4.4.2 3D Simple Vision Transformer

In contrast to CNNs, which combine local image information using convolutional filters, vision transformers (Dosovitskiy et al. 2021) process images through a different mechanism. Essentially, vision transformers divide the input image into a sequence of image patches, then combine information across these patches to characterize visual features. Since all image patches are connected via a so‐called attention mechanism (Vaswani et al. 2017), vision transformers can generate visual features composed of spatially unrelated information in the input image. In comparison, CNNs are limited to combining information from local image neighborhoods to form visual features.

We adapted a sViT (Beyer et al. 2022) to predict age from 3D sMRI scans. A brief description of the specific modifications is given in Appendix A. The 3D sViT implementation we used can be found in the GitHub repository vit‐pytorch^4^. Hyperparameters were kept at the vit‐pytorch defaults. The complete set of hyperparameters is shown in Table A1, resulting in a total of 42.0 million trainable parameters.

#### 4.4.3 3D Shifted Window Transformer

The SwinT (Liu et al. 2021) is a modification of the original vision transformer (Dosovitskiy et al. 2021), which reintroduces core properties of CNNs to improve performance on visual tasks. Like the vision transformer, the SwinT divides input images into image patches. However, it focuses on forming visual features by combining information from locally related image patches, while distant image patches are only connected via indirect pathways. This modification means that the SwinT loses some of the vision transformer's flexibility in creating visual features, but large images in particular can be processed more efficiently. In addition, the SwinT fuses image patches at different levels of depth, which makes the SwinT learn hierarchical image representations, which are crucial for biological vision (Hubel and Wiesel 1962), and fundamental to CNNs (LeCun et al. 2010).

Similar to the sViT, we adapted the SwinT to operate on 3D input (Appendix A). Our implementation and hyperparameter choices regarding the number of attention heads, patch size, embedding dimension, and attention window size were based on the SwinUNETR model (Hatamizadeh et al. 2021), previously used for 3D brain tumor segmentation. The model depth and the expansion ration of the multilayer perceptron α were based on the “Swin‐T” model variant from Liu et al. (2021). All hyperparameters are detailed in Table A2, resulting in 10.1 million trainable parameters.

### 4.5 Model Training

All model architectures were trained using the PyTorch Lightning 1.8 interface for PyTorch 1.12 and a single Nvidia A100 GPU with 80GB memory for ResNet and sViT, and two A100s of the same type for the SwinT. Each model was optimized using Adam (Kingma and Ba 2014) on the mean squared error loss, with a one‐cycle learning rate policy (Smith and Topin 2019; Fisch et al. 2021; Schulz et al. 2022). The maximum learning rate for SwinT and sViT was set to $10^{-4}$ and to $10^{-2}$ for the ResNet. The training duration was 150,000 gradient update steps for each model architecture. The effective batch size was 8 for ResNet and SwinT, and 16 for sViT. Each model architecture was re‐trained 6 times with different random initialization and batch order.

### 4.6 Measuring Clinical Utility

We evaluated clinical utility of BAGs through two measures: their sensitivity to neurological and mental diseases (AD, PD, MS, depression, schizophrenia, BD), and their predictive power for health‐related phenotypes (fluid intelligence, reaction time, trail making interval, tobacco consumption, mobile phone usage, TV consumption, systolic blood pressure, grip strength, and BMI). Our analysis workflow proceeded as follows (see Figure 1 for an overview): First, we trained multiple instances of sViT, SwinT and ResNet using the normative cohort. Second, we computed BAGs of held‐out patients and controls by subtracting chronological age from predicted age, for each of the models' instances. Third, we quantified BAG sensitivity to diseases by calculating effect sizes (Cohen's *d*) between patient and matched control BAGs, with effect size uncertainties estimated via bootstrapping across patient‐control pairs. Finally, we assessed BAGs' predictive power for health‐related phenotypes by fitting linear models that included BAGs and covariates (age, sex, genetic principal components 1‐3, years of education, income level) as predictors. For each phenotype, we report statistics of the BAG's 𝛽‐coefficient as a measure of its predictive strength, and estimated uncertainties via bootstrapping.

### 4.7 Measuring Consistency of Brain Age Concepts Across Train Runs

Brain age concepts may vary not only due to differences in model architecture, but also due to random weight initialization and batch order during training. To examine this variation, we trained 6 instances of each model architecture with varying initializations and batch orders. We then analyzed prediction correlations across model instances for held‐out patients and controls using Pearson's correlation coefficient. This analysis helped quantify potential differences in brain age concepts arising from different training runs.

## 5 Results

### 5.1 SwinT Is Competitive and Will Likely Outperform ResNet With Increasing Sample Sizes

To investigate whether transformers may outperform CNNs in accurately predicting brain age, we compared mean absolute errors (MAEs) for held‐out healthy subjects between SwinT, sViT, and ResNet. SwinT (MAE of 2.67 ± 0.02, mean and SD over different train runs) and ResNet (MAE of 2.66 ± 0.05) performed on par (Table 1). sViT performed noticeably worse, with an averaged MAE of 3.02 ± 0.08 years.

**TABLE 1:** SwinT achieves ResNet‐level accuracy in brain age prediction.

Model	Test MAE (years)	Test *R* ^2^
ResNet	**2.66 ± 0.05**	**0.81 ± 0.01**
SwinT	2.67 ± 0.02	**0.81 ± 0.00**
sVit	3.02 ± 0.08	0.76 ± 0.01

*Note:* Mean absolute errors (MAEs) and coefficient of determination ($R^{2}$) are displayed for the held‐out set of healthy subjects (n=1172). The uncertainty estimates indicate the standard deviation (SD) across different randomly initialized model instances. 3D ResNet50 (ResNet) and 3D shifted window transformer (SwinT) predict age with nearly identical accuracy, both outperforming the 3D simple vision transformer (sViT). Bold indicates the best‐performing model architecture.

**FIGURE 2:**
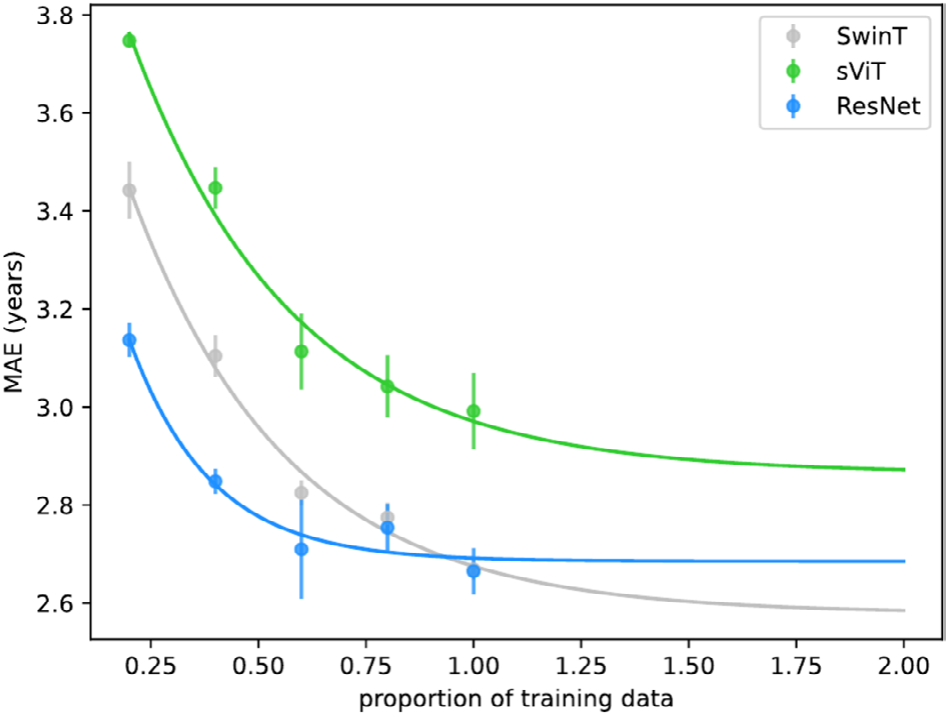
SwinT will likely to outperform ResNet with additional training samples We trained multiple instances of each model architecture with gradually decreased training samples and found that accuracies of shifted window transformer (SwinT) and simple vision transformer (sViT) decline stronger compared to the ResNet. Extrapolating each model architecture's accuracy using power laws (Schulz, Bzdok, et al. 2024) indicates SwinT would surpass ResNet's accuracy given additional training samples. Uncertainty estimates refer to the SD across model instances.

We further examined each architecture's accuracy scaling with training sample size. By training model instances on progressively reduced datasets and applying power‐law scaling relations (Schulz, Bzdok, et al. 2024), we could extrapolate expected accuracies beyond available training data. We found that the SwinT can be expected to outperform the ResNet starting from approximately n=25,000 samples (Figure 2), with the ResNet marginally benefitting from more training samples. The sViT's performance can be expected to benefit from increasing training samples, though it may not be able to achieve accuracies comparable to SwinT and ResNet in its current form.

### 5.2 No Evidence That sViT, SwinT, and ResNet Attend to Different Concepts of Brain Age

To investigate whether SwinT, sViT and ResNet may attend to different concepts of brain age, we analyzed differences in predictions and prediction errors as proxies of differences in the underlying aging characterizations (see Section 3.3). In a first analysis, we computed the Pearson correlation for held‐out‐set predictions between model architectures. Predictions from all three model architectures were highly correlated (average correlation with SD between predictions of differently initialized SwinT and ResNet instances: $\bar{r}=0.94\pm 0$, SwinT‐sViT: $\bar{r}=0.91\pm 0.03$, ResNet‐sViT: $\bar{r}=0.91\pm 0.1$), suggesting that each model architecture follows a similar concept of brain age.

**FIGURE 3:**
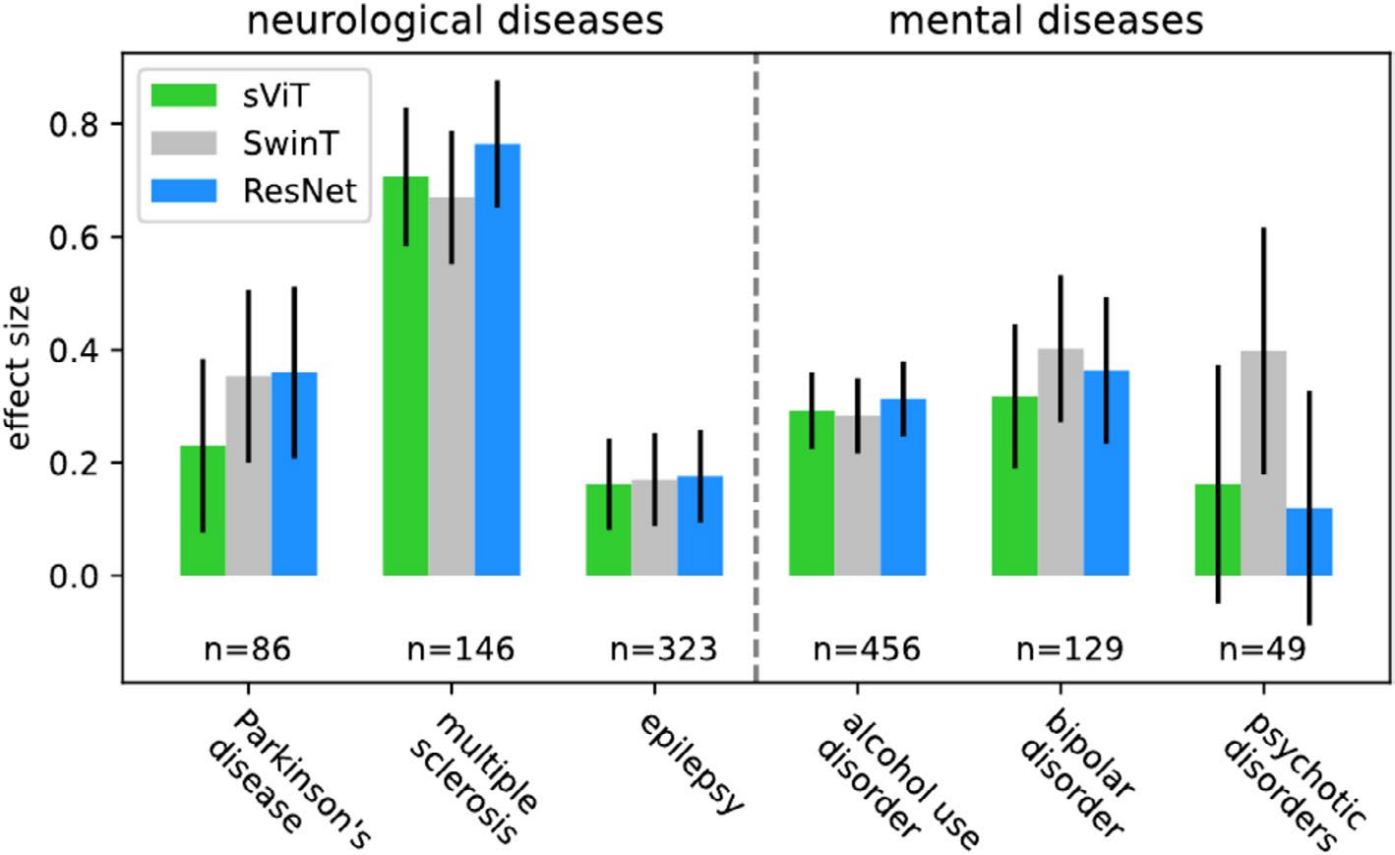
Different brain age model architectures encode similar disease patterns. The figure shows effect sizes (Cohen's d) measured between BAGs of patients and matched controls. Effect sizes between model architectures were within one σ from each other for any disease, with no indication of differences. Error bars indicate the standard error of the mean estimate derived by bootstrapping patient‐control pairs.

**FIGURE 4:**
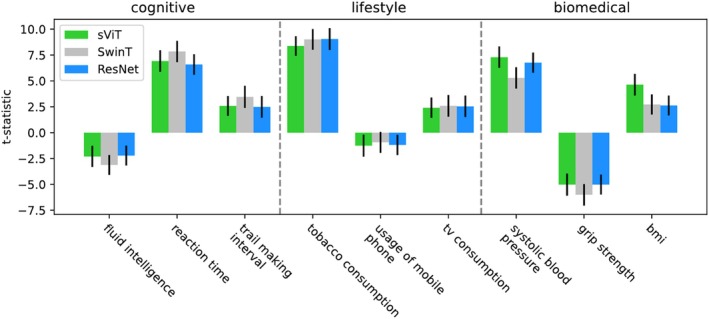
Association of BAG and cognitive, lifestyle and biomedical phenotypes seems not to depend on the model architecture. We fitted linear models from BAG and confounds to phenotype and report the t‐statistic for whether the BAG is a significant predictor. Error bars indicate the t‐statistic's standard error of the mean estimate, derived by bootstrapping. BAGs of different model architectures were similarly predictive for the analyzed phenotypes.

In a second analysis, we compared the clinical utility (Section 3.2) of each model architectures' BAGs. Deviations in clinical utility between model architectures would hint to differences in the concepts of brain age (see Section 3.3). We found that the sensitivity of BAGs for the investigated disorders were comparable across model architectures. Patients' BAGs were elevated for each model architecture and disease (Figure 3). We observed (in Cohen's terminology; Cohen 2013) small effects for epilepsy, small to medium effects for PD, AUD, BD and psychotic disorders, and medium to large effects for MS. Effect sizes between model architectures were within one σ from each other for any disease, with no indication of differences. The association of BAG and cognitive, lifestyle, and biomedical phenotypes was also comparable across model architectures. Again, the measured effects were within approximately one σ from each other, again with no indication of a difference (Figure 4). The size and directionality of effects was compatible with literature expectations: Weak results on cognitive tests, unhealthy habits, and markers of poor physical condition were associated with elevated BAG, while good results on cognitive tests and markers of good physical condition were associated with a decreased BAG (Smith et al. 2019).

**FIGURE 5:**
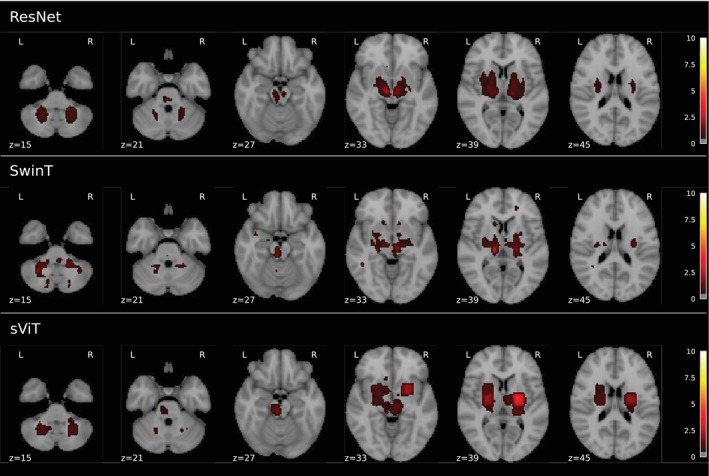
Similar brain features appear to be relevant for age predictions across different model architectures. Using Input × Gradient (IxG) Shrikumar et al. (2016), we generated feature‐relevance heatmaps for each held‐out healthy subject across ResNet, SwinT, and sViT. These heatmaps, averaged across random model architecture initializations and visualized at group‐level using a color scale (dark red = low relevance, white = high), revealed highly consistent brain regions across architectures, suggesting they capture comparable features of brain aging. Slight variations in the heatmaps likely stem from interactions between the model architectures and IxG, rather than reflecting meaningful differences in the underlying relevant features. The consistency in highlighted brain regions across ResNet, SwinT and sViT reinforces our conclusion that different model architectures are unlikely to learn different concepts of brain age. Notably, brain regions such as the cerebellum, basal ganglia, and brain stem, which were consistently identified as important, are well‐documented for their roles in aging processes (Walhovd et al. 2011), further validating their relevance as predictors of age.

A supplementary analysis examined feature relevance across model architectures using InputxGradient (IxG) (Shrikumar et al. 2016) visualization (Figure 5, details in Appendix B). Feature‐relevance heatmaps were generated for each architecture using held‐out healthy subjects. Group‐level analysis revealed consistent patterns across SwinT, sViT, and ResNet, highlighting aging‐sensitive regions including the cerebellum, basal ganglia, and brain stem—areas previously established as primary aging targets (Walhovd et al. 2011). The convergent spatial patterns suggest shared feature relevance across architectures. Minor visual differences in the heatmaps likely reflect methodology‐specific interactions between architectures and IxG rather than fundamental differences in feature importance.

In summary, our investigations provide no evidence that sViT, SwinT, and ResNet focus on different concepts of brain age. Across model architectures, we observed: (1) highly correlated age predictions, (2) comparable BAG sensitivity to neurological and psychiatric conditions, (3) consistent BAG associations with cognitive, lifestyle, and biomedical phenotypes, and (4) similar patterns of feature relevance in age prediction. Together, these complementary findings suggest that different model architectures are unlikely to learn meaningfully different concepts of brain age.

### 5.3 Concepts of Brain Age Appear Consistent Across Train Runs

To assess the consistency of either model architecture's brain age concept across random initializations and batch orders, we computed correlations between held‐out‐subject predictions within each model architecture and found no indication of varying brain age concepts. Over six different train runs, SwinT averaged a Pearson correlation of $\bar{r}=0.98\pm 0.01$ (SD) ($\bar{r}=0.96\pm 0.02$ for ResNet; $\bar{r}=0.94\pm 0.03$ for sViT), suggesting that brain age concepts are mostly unaffected by random initializations and batch order. In comparison to sViT and ResNet, the SwinT appears to converge to more uniform brain age concepts.

## 6 Discussion

In the present study, we make three central contributions. First, we adapt and evaluate the recently popularized transformer architecture for brain age prediction. Using one of the largest brain imaging datasets currently available, we demonstrate that the novel SwinT and the widely used ResNet predict age with nearly identical accuracy. Our results indicate that both evaluated transformer architectures will benefit from growing sMRI datasets, while the accuracy of ResNet appeared to be saturated. Second, we identify that “brain age” might not refer to a uniform concept and outline why “concepts of brain age” may differ between brain age models. Third, we investigate whether conceptually different deep learning model architectures attend to different concepts of brain age. Through extensive analysis of structural differences in brain age predictions under a range of neurological and psychiatric disorders, and their associations with biomedical, cognitive, and behavioral phenotypes, we find no indication that SwinT, ResNet, and sViT attend to different concepts of brain age.

### 6.1 Transformers for Accurate Brain Age Prediction

We evaluated two of the most popular vision transformers for brain age prediction and found that the SwinT achieves comparable performance to the widely used ResNet CNN (ResNet MAE 2.66 years, SwinT MAE 2.67), with evidence suggesting superior performance at larger sample sizes (Figure 2). Our model architectures' performance falls within the competitive range of previously reported CNNs trained on UKBB data (MAEs: 2.14‐2.86 years; (Tanveer et al. 2023)). While lower MAEs have been reported (2.14; Peng et al. 2021), these results rely on performance‐enhancing measures such as ensembling, data augmentation, and label binning, which we excluded to maintain the generalizability of our model architecture comparison.

As the number of available sMRI images in large databases like the UKBB continues to grow, the SwinT, given its scaling performance in Figure 2, is likely to replace the ResNet as the de facto default deep learning model architecture for brain age prediction.

### 6.2 Potentially Different Concepts of Brain Age Between Model Architectures

The use of different model architectures in brain age prediction risks unknowingly researching different concepts of brain age, which would raise several concerns. First, prior findings on the clinical utility of BAGs as biomarkers could be confounded by the choice of model architecture. Second, selecting a model architecture for brain age prediction would necessitate evaluating the clinical utility of BAGs rather than relying solely on model accuracy. Third, architecture‐specific BAGs could potentially reflect distinct diseases, challenging the role of BAG as a general brain health biomarker. These considerations raise a crucial question: do distinct model architectures focus on different concepts of brain age, potentially leading to different biomarkers?

Our analyses provide reassurance, as we found no evidence that model architectures consider meaningfully different concepts of brain age (Section 5.2). This finding mitigates concerns about model architecture confounding in deep brain age studies and indicates that the clinical utility of BAGs remains independent of the chosen deep learning model architecture. Consequently, clinical utility need not be considered when selecting a model architecture. Furthermore, our results suggest that research aimed at generating BAGs with increased clinical utility should focus on factors other than model architecture selection (see Section 6.4). Importantly, we found no evidence of architecture‐specific disease biomarkers, supporting the use of BAG as a general brain health biomarker, as previously suggested by Cole and Franke (2017).

Given the conceptually distinct model architectures analyzed, which we consider the most plausible cause of potential variations in brain age concepts, we believe that our results should generalize to related model architectures, such as various CNNs used in previous brain age studies (Peng et al. 2021; Huang et al. 2017; Kolbeinsson et al. 2020).

### 6.3 Potentially Different Concepts of Brain Age Across Train Runs

The potential influence of random factors such as weight initialization and batch order during training raises concerns about brain age concept stability, particularly since many brain age studies rely on single model instances (Bashyam et al. 2020; Cole et al. 2017; Jónsson et al. 2019). However, our results suggest stability in brain age concepts across different training runs for sViT, ResNet, and SwinT architectures. Compared to model architecture potentially confounding brain age studies, issues related to random influences are less problematic because ensembling could be used to account for any variance within a model architecture.

### 6.4 Relation Between Deep Learning Model Architecture's Accuracy and Clinical Utility

Our results suggest that clinical utility and deep learning model architecture are unrelated, however, we also found that the noticeably less accurate sViT generated BAGs with very similar clinical utility to BAGs of the more accurate SwinT and sViT, which contrasts with the common belief that more accurate models lead to more useful biomarkers (Hahn et al. 2021; Peng et al. 2021; Cole 2020; Tanveer et al. 2023; Niu et al. 2020). Along the same lines, previous work has questioned the relation between accuracy and clinical utility: Bashyam et al. (2020) have reported that stopping CNNs' training before convergence increases biomarker utility; Jirsaraie et al. (2023) have reviewed multimodal brain age studies, including deep and traditional ML models, and have not found a relation between accuracy and clinical utility; Schulz, Siegel, et al. (2024) have shown that simple linear models, yielded more useful biomarkers than their more accurate deep counterparts. This growing body of evidence, combined with our findings, suggests that optimizing model architectures for prediction accuracy may not be the optimal approach to generating useful biomarkers. Research focusing on training protocol modifications, such as early stopping (Bashyam et al. 2020) and overregularization (Schulz, Siegel, et al. 2024), appears more promising.

### 6.5 Questionable Construct Validity of Brain Age

While our study found no evidence that model architecture, weight initialization, or batch order affects concepts of brain age, Schulz, Siegel, et al. (2024) demonstrated that reducing model expressivity through overregularisation can produce distinct concepts of brain age. These constrained models, despite lower age prediction accuracy, appear to achieve superior clinical utility. Other work questions whether individual differences in brain age relate to aging effects at all: Vidal‐Pineiro et al. (2021) argue that birth‐weight and genetic factors have a greater impact on BAGs than actual longitudinal brain change. These findings challenge the construct validity of the brain‐age gap, highlighting the need for more precise terminology and methodology to investigate and characterize the underlying concepts of brain age learned by machine learning algorithms.

### 6.6 Limitations

Our study has three important limitations. First, computational constraints prevented optimization of the transformers for prediction accuracy. Training a single instance of either transformer model architecture required multiple days on the available GPUs, and the hyperparameter configuration space is vast, making thorough optimization impractical. As a result, the reported accuracies should be considered promising lower bounds to optimized accuracies rather than precise estimates. Future research should focus on optimizing the SwinT's brain age prediction accuracy.

Second, while we argue against differences in brain age concepts between model architectures, proving such absence presents inherent challenges. It is virtually impossible to exhaustively test all conditions (architectures, hyperparameters, demographic factors) that could reveal conceptual differences. Nevertheless, our findings remain informative due to our careful selection of architectures based on fundamental design differences—the most likely source of brain age concept variation. Also, we examined a broad set of demographic factors with various neural correlates (Raz et al. 2005; Geng et al. 2006; Gallinat et al. 2006; Dekker et al. 2021; Gómez‐Apo et al. 2021), which in its entirety should provide comprehensive sensitivity to meaningful differences in brain age concepts.

Third, we focused on analyzing predictions and prediction errors' clinical utility as proxies for the underlying concepts of brain age, using explainable artificial intelligence (XAI) only as supplementary evidence. While more extensive XAI applications—such as analyzing feature‐relevance maps for patient subgroups or incorporating additional methods—could offer deeper insights, we limited this approach due to ongoing concerns about XAI reliability. These concerns include common XAI methods' failure to satisfy key theoretical axioms (Sundararajan et al. 2017), inability to outperform random relevance assignments (Hooker et al. 2019), the production of heatmaps that can be independent of model parameters or training data (Adebayo et al. 2018), and susceptibility to imperceptible input perturbations (Ghorbani et al. 2019; Kindermans et al. 2019; Dombrowski et al. 2019).

Given these limitations, validating XAI methods' reliability for explaining neuroimaging deep learning predictions is essential before drawing major conclusions. To our knowledge, no such validation has been conducted on brain imaging data. This is partly because XAI validation often defaults to visual inspection (Doshi‐Velez and Kim 2017), yet expectations for explanation maps in the neuroimaging field are often a priori unknown or highly difficult to characterize.

### 6.7 Conclusion

In this study, we highlight the possibility of heterogeneity in “concepts of brain age” learned by modern machine learning algorithms.

Reassuringly, we found no indications that deep learning model architectures attend to different concepts of brain age, and hence, it appears unlikely that previous deep brain age studies' results, for example regarding the clinical utility of BAGs, have been confounded by the model architecture used.

## Author Contributions

**Nys Tjade Siegel:** data curation, methodology, software, formal analysis, visualization, writing – original draft. **Dagmar Kainmueller:** formal analysis, methodology, writing – review and editing. **Fatma Deniz:** formal analysis, methodology, writing – review and editing. **Kerstin Ritter:** conceptualization, methodology, formal analysis, writing – review and editing, funding acquisition. **Marc‐Andre Schulz:** conceptualization, methodology, software, formal analysis, writing – original draft, writing – review and editing, supervision.

## Supporting information


**Table S1.** Mappings from diseases (first occurrence dates) to UK Biobank field numbers.
**Table S2.** Mappings from variable names to UK Biobank field numbers.

## Data Availability

Data are available from the UK Biobank upon request.

## Endnotes

^1^
We distinguish our use of the term “concept” from other ML literature, in which the term concept is sometimes associated with something explicitly humanly understandable. Here, we refer to a deep learning model's notion of brain age in terms of the distinct aging effects exploited. Notably, the human understanding of brain age is itself ambiguous, lacking a clear definition of which aging effects contribute to “brain age”. Instead, brain age is only implicitly defined through a ML model's notion of brain age.

^2^
Psychotic disorders refer to schizophrenia, schizotypal and delusional disorders (ICD‐10 codes F20 to F29). They are treated as a single group in our analysis due to the impractically small sample sizes (n<33) when treated separately.

^3^

https://github.com/kenshohara/3D‐ResNets‐PyTorch.

^4^

https://github.com/lucidrains/vit‐pytorch.

## Appendix A Adapting Vision Transformers to Predict Age From 3D sMRI Scans

We adapted both SwinT and sViT to operate on 3D input, by dividing input images into 3D image cubes, instead of 2D image patches, aligning with previous efforts that adjusted transformers for 3D MRI data Hatamizadeh et al. (2021); Jiang et al. (2022); Peiris et al. (2022); Wu et al. (2023); Karimi et al. (2021). Also, we used sinusoidal positional encodings (Vaswani et al. 2017) in the SwinT, in addition to the relative position bias present by default. Sinusoidal positional encodings provide information on an image cubes' absolute positions in the input image. We anticipated that information on absolute cube position would benefit the model architectures, given that we linearly registered input images to the MNI152 reference space, which leads to image cubes displaying very similar brain regions across subjects. Similarly, sinusoidal positional encodings were employed in the sViT as part of its default setting. Finally, we applied linear regression layers after the transformer‐based encoders, to obtain scalar age predictions.

**TABLE A1:** Hyperparameters for the 3D sViT.

Layers	6
Heads per layer	8
Patch size	16, 16, 16
Embedding size	1024
MLP size	2048

**TABLE A2:** Hyperparameters for the 3D SwinT.

SwinT blocks per stage	2, 2, 6, 2
Attention heads in blocks of each stage	3, 6, 12, 24
Patch size	2, 2, 2
Attention window size	4, 4, 4
Initial embedding size	48
MLP expansion factor (α)	4

## Appendix B Visualizing Relevant Brain Features for Age Prediction Across Model Architectures

Visualizing the brain features that different model architectures consider important for age predictions could provide insights into whether these architectures attend to different concepts of brain age. Such a visualization can be done by obtaining feature‐relevance heatmaps using methods from XAI. These feature‐relevance heatmaps indicate which parts of the input have been relevant to ML models' predictions on single‐subject level.

To visualize which brain features were relevant to age predictions by SwinT, sViT, and ResNet, we generated feature‐relevance heatmaps using IxG (Shrikumar et al. 2016), which has a clear theoretical justification and is applicable to transformers and CNNs in the same way despite their architectural differences. We computed heatmaps for each held‐out healthy subject, model architecture, and random model architecture initialization. To manage memory consumption, we downsampled the heatmaps (local mean downsampling to half resolution) and applied a brain mask. Subsequent postprocessing steps included taking absolute heatmap values, scaling heatmaps to their 99th percentile for comparability across architectures and initializations, and averaging across subjects to produce group‐level heatmaps. These group‐level heatmaps were further smoothed using a 3D Gaussian filter (full‐width at half maximum = 2 mm) in unmasked image space. To highlight features driven by the model architecture rather than random noise, we then averaged the heatmaps across random initializations for each model architecture. Finally, we displayed each resulting heatmap with a cutoff of 0.5 to ensure easy visual accessibility.

The resulting heatmaps, shown in Figure 5, revealed consistent important brain regions across model architectures, suggesting that similar brain features have been important for age predictions across the model architectures, supporting our conclusion that different model architectures are unlikely to attend to meaningfully different concepts of brain age.
