# Supplementary figures and images for: Do Transformers and CNNs Learn Different Concepts of Brain Age?
Source: Hum Brain Mapp. 2025 Jun 9;46(8):e70243. doi: 10.1002/hbm.70243 (PMC12147945; doi:10.1002/hbm.70243)

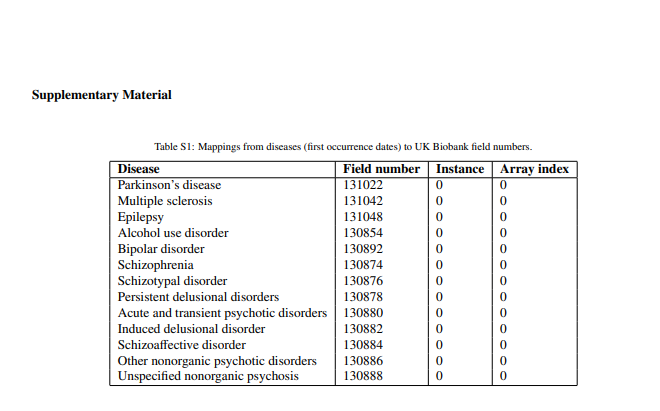


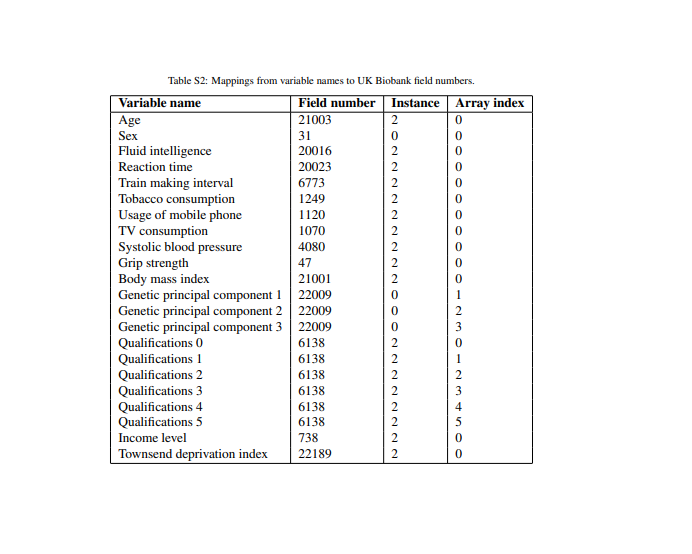

Supplement: Supplementary file 1 — Table S1. Mappings from diseases (first occurrence dates) to UK Biobank field numbers. Table S2. Mappings from variable names to UK Biobank field numbers. [file HBM-46-e70243-s001.docx]
